# Use of Oral and Emergency Contraceptives After the US Supreme Court’s *Dobbs* Decision

**DOI:** 10.1001/jamanetworkopen.2024.18620

**Published:** 2024-06-26

**Authors:** Dima M. Qato, Rebecca Myerson, Andrew Shooshtari, Jenny S. Guadamuz, G. Caleb Alexander

**Affiliations:** 1 Program on Medicines and Public Health, Alfred Mann School of Pharmacy and Pharmaceutical Sciences, University of Southern California, Los Angeles; 2 Leonard D. Schaeffer Center for Health Policy and Economics, University of Southern California, Los Angeles; 3 Department of Population Health Sciences, University of Wisconsin, Madison; 4 Division of Health Policy and Management, University of California at Berkeley School of Public Health, Berkeley; 5 Department of Epidemiology at the Johns Hopkins Bloomberg School of Public Health, Baltimore, Maryland; 6 Center for Drug Safety and Effectiveness, Johns Hopkins Bloomberg School of Public Health, Baltimore, Maryland

## Abstract

**Importance:**

The US Supreme Court *Dobbs v Jackson Women’s Health Organization* decision allowed states to strengthen restrictions on abortion access, triggering the closure of family planning clinics and leading to confusion about the legality of emergency contraceptives (ECs).

**Objectives:**

To evaluate the association between the *Dobbs* decision and fills for oral and emergency contraceptives in states that enacted the most restrictive abortion policies after *Dobbs.*

**Design, Setting, and Participants:**

This cohort study used data on contraceptive fills for women of reproductive age (15-49 years) in the US from IQVIA’s National Prescription Audit PayerTrak and data from the Guttmacher Institute were used to categorize changes in abortion restrictions in each state. A difference-in-differences analysis compared changes in monthly fill rates for daily oral contraceptive pills (OCPs) and ECs in states that became most restrictive (implemented a full abortion ban after *Dobbs*) and comparison states (kept a medium level of abortion restrictions after *Dobbs)* before (March 2021 to November 2021) and after (July 2022 to October 2023) the *Dobbs* decision.

**Exposure:**

State-level abortion restrictions.

**Main Outcomes and Measures:**

Monthly fills of OCPs and ECs per 100 000 women of reproductive age.

**Results:**

Between March 2021 and October 2023, 142.8 million prescriptions for OCPs and 904 269 prescriptions for ECs were dispensed at US retail pharmacies. Before *Dobbs*, trends in monthly fill rates were similar for OCPs and ECs between the most restrictive and comparison states. After the *Dobbs* decision, states that became the most restrictive experienced an additional 4.1% decline in OCP fills with 285.9 fewer fills per 100 000 (95% CI, −495.8 to −6.8; *P* = .04). In contrast to OCPs, fills for ECs increased during the first year after *Dobbs* (July 2022 to June 2023) in both groups of states. However, 1 year after *Dobbs* (July 2023 to October 2023), the most restrictive states experienced an additional 65% decrease in emergency contraceptive fills with 13.2 fewer fills per 100 000 (95% CI, −27.2 to −4.1; *P* = .01).

**Conclusions and Relevance:**

In this cohort study of prescriptions filled at US pharmacies, the *Dobbs* decision was associated with declines in oral contraceptives, particularly ECs, in states enacting the most restrictive abortion policies. Given the important role of OCPs and ECs in preventing pregnancy and the need for abortion, efforts to improve access may be needed, especially in states where legal abortion is no longer an option.

## Key Points

**Question:**

What is the association between the US Supreme Court’s July 2022 *Dobbs v Jackson Women’s Health Organization* decision and fills for oral and emergency contraceptives?

**Findings:**

In this cohort study of over 143 million prescriptions dispensed at US retail pharmacies from March 2021 through October 2023, the *Dobbs* decision was associated with declines in fills for oral contraceptives—both daily oral contraceptive pills and emergency contraceptives—in states that implemented the most restrictive policies with a full ban on abortion. One year after *Dobbs*, declines were greatest for emergency contraceptives; states that became the most restrictive experienced an additional 65% decrease in fills for emergency contraceptives compared with states that kept moderate levels of abortion restrictions after *Dobbs*.

**Meaning:**

These findings suggest that efforts to protect and improve access to oral contraceptives are needed, especially for emergency contraceptives in states where abortion is most strongly restricted.

## Teaser

This cohort study evaluates the association between the *Dobbs v Jackson Women’s Health Organization* decision and fills for oral and emergency contraceptives in states that enacted the most restrictive abortion policies after the decision*.*

## Introduction

Oral contraceptives are critical in preventing pregnancy and the need for abortion^1,2,3^ and are the most commonly used and preferred method of hormonal contraception in the US.^4,5^ Oral contraceptives have also been at the center of longstanding legal and policy efforts to both expand as well as restrict access to contraception,^6,7,8,9^ including for emergency contraceptives (ECs).^10,11^ The US Supreme Court decision in the *Dobbs v Jackson Women’s Health Organization*, which overturned *Roe v Wade* on June 24, 2022,^13^ may have also influenced access to contraception.^14^ Since the *Dobb*s decision, 26 states enacted full or partial bans on abortion,^15^ and there are concerns that some may also strengthen restrictions on access to contraception, particularly ECs.^16,17,18^ In fact, in response to concerns that some states may misinterpret abortion bans to include ECs as abortifacients, the Food and Drug Administration updated product labeling for levonorgestrel to clarify it does not affect fertilization or implantation or terminate pregnancy.^19^

Prior work has investigated the impact of federal and state restrictive abortion policies on access to contraception.^20,21,22,23,24^ For example, the 2019 Title X Final Rule, which prohibited participating family planning clinics from offering abortion services, reduced the availability of Title X clinics, the provision of contraception services, and the use of contraceptives.^20,21,22^ The implementation of restrictive abortion and family planning policies in Texas between 2011 and 2014 led to the closure of publicly supported clinics and increases in abortion rates with minimal impact on over-the-counter purchases of ECs (ie, levonorgestrel) or condoms.^23^ In Iowa, 2 years after the state imposed Medicaid coverage restrictions on publicly supported family planning clinics providing abortion services, the use of contraceptives declined by two-thirds.^24^

Despite these insights, the association between the recent *Dobbs* decision and the use of oral contraceptives—both daily oral contraceptive pills (OCPs) and ECs—is not known. This information is important because many family planning clinics closed directly following the *Dobbs* decision, particularly in states that became most restrictive with a full ban on abortion.^25,26^ These closures may have reduced access to contraceptives because 11% of women rely on these clinics for their contraceptive care,^10^ including prescriptions for oral contraceptives.^27^ The *Dobbs* decision may have also contributed to declines in the use of ECs due to misunderstanding about their legality, particularly in restrictive states.^17^ In fact, a January 2023 survey found that half of women in full ban states believed levonorgestrel was illegal in their state.^18^

We quantified trends in prescription fills for oral contraceptives—both daily OCPs and ECs—using data on fills in US retail pharmacies between March 2021 and October 2023. We compared trends in fills for oral contraceptives across groups of states that varied in their changes in abortion policy after *Dobbs*, and evaluated the association between the *Dobbs* decision and oral contraceptive fills in states that implemented the most restrictive policies with a full ban on abortion. We hypothesized that *Dobbs* would be associated with declines in the fills for oral contraceptives, particularly ECs, in states implementing the most restrictive abortion policies.

## Methods

### Data and Setting

We used data from 3 sources. First, we used IQVIA’s National Prescription Audit (NPA) and PayerTrak to estimate monthly volume for prescriptions for oral contraceptives dispensed at pharmacies at the national and state level, respectively. These data represent projected estimates using information from more than 93% of retail pharmacies in the US,^13^ and capture information on prescription characteristics, including prescriber specialty (eg, nurse practitioner, obstetrics and gynecology) and method of payment (eg, Medicaid, commercial, or paid in cash). The study was determined by the University of Southern California institutional review board to not constitute human participant research and was waived from review and the need for informed consent. This study followed the Strengthening the Reporting of Observational Studies in Epidemiology (STROBE) reporting guidelines.

We defined daily OCPs using the Anatomic Therapeutic Categories (ATC) (G03A1, G03A2, G03A3, G03A4, and G03A5), and we defined oral ECs using ATC G03A6, which includes ulipristal and levonorgestrel. We focused on oral contraceptives because they are the most used hormonal method of contraception filled at retail pharmacies (eFigure 1 in Supplement 1). Specifically, 11.5% of women used OCPs filled at retail pharmacies in 2019, which is an estimated 85% of women that use OCPs overall (eTable 1 in Supplement 1). In contrast, long-acting reversible contraceptives (LARCs), such as intrauterine devices, injectables, and implants, are not self-administered and are nearly always dispensed in clinics rather than retail pharmacies.

Second, we used the 2021 American Community Survey (ACS) to derive the total population of women and girls of reproductive age (15 to 49 years), nationally and for each state. Third, we used data from the Guttmacher Institute to classify each state based on changes in policies related to abortion restrictions and protections between June 2022 (the month of the *Dobbs* decision) and October 2023.^18^ The Institute assesses a range of state policies related to abortion based on approximately 20 types of restrictions, such as gestational age bans, waiting periods, insurance coverage bans, and medication abortion limits, as well as 10 protective policies, such as state constitutional protections, abortion funding, insurance coverage for abortion, and protections for patients and clinic staff. States are then assigned to 1 of 7 categories (most restrictive, very restrictive, restrictive, some restrictions, protective, very protective, and most protective) with monthly updates (eTable 2 in Supplement 1). We classified states into 6 abortion policy categories based on changes (if any) after *Dobbs* as follows: (1) became most restrictive shortly after *Dobbs* (ie, by August 2022); (2) became more restrictive after *Dobbs*; (3) no change after *Dobbs*, remained restrictive; (4) no change after *Dobbs*, some restrictions; (5) no change after *Dobbs*, remained protective and (6) became more protective after *Dobbs* (eTable 3 in Supplement 1).

### Outcome Measures

We calculated monthly rates of prescription fills per 100 000 women of reproductive age for daily OCPs and oral ECs (primary outcomes) and for ulipristal and levonorgestrel separately (secondary outcomes) nationally and for each state between March 2021 and October 2023. We analyzed ulipristal and levonorgestrel separately because ulipristal is prescription-only whereas levonorgestrel is available over-the-counter, although many private and Medicaid insurance plans provide coverage without cost-sharing when levonorgestrel is filled with a prescription.^8^

### Statistical Analyses

We first conducted a descriptive analysis to characterize prescriptions filled at retail pharmacies for oral contraceptives by pharmacy type, prescriber specialty, and method of payment between March 2021 and October 2023. We then analyzed monthly rates for each outcome measure nationally, by state, and by state abortion policy category between March 2021 and October 2023.

Women may have changed their contraceptive choices after the oral arguments of *Dobbs* (ie, in anticipation of coming changes in their state’s abortion policy) or after *Dobbs* (ie, in response to changes in their state’s abortion policy). The *Dobbs* decision had oral arguments on December 1, 2021, and the decision was formally announced on June 24, 2022. Accordingly, we extracted monthly rates in the following periods: before the *Dobbs* oral arguments (March 2021 to November 2021), after the oral arguments were made but before the *Dobbs* decision was announced (December 2021 to June 2022), the first year after the *Dobbs* decision (July 2022 to June 2023), and more than 1 year after the *Dobbs* decision (July to October 2023). We separately assessed changes during the first year after *Dobbs* and changes after more than 1 year because *Dobbs* may have had a differential impact earlier (eg, stockpiling of ECs) and prescriptions for OCPs cannot have a duration longer than 12 months.

To determine the association between states enacting strong abortion restrictions after the *Dobbs* decision and changes in oral contraceptive use, we used a difference-in-differences (DID) design. This analysis compared changes in oral contraceptive fills before and after the *Dobbs* decision in states that enacted the strongest level of abortion restrictions shortly after the *Dobbs* decision (most restrictive with a nearly full ban on abortion) vs a group of comparison states that did not change their level of restrictiveness after *Dobbs* (states that remained restrictive or remained with some restrictions after *Dobbs*). The most restrictive group included 12 states, and the comparison group included 14 states. The DID estimates were calculated using linear regression models that adjusted for trends by calendar month and state; models weighted the data from each state based on the size of the population of women of reproductive age. Standard errors were clustered by state, using the wild cluster bootstrap procedure due to the small number of clusters. The plausibility of the parallel trends assumption was examined by testing whether trends in fills from March 2021 through November 2021 (pre-*Dobbs*) differed between the most restrictive and comparison states.

To explore the association between these post-Dobbs changes in abortion policy and fills for oral contraceptives, we also analyzed trends in monthly fills for OCPs and ECs for states with different changes in abortion policy before and after *Dobbs.* We calculated *P* values using 2-sided tests with statistical significance set at *P* < .05. Analyses were conducted using Stata statistical software, version 18.0 (StataCorp).

We conducted 2 sensitivity analyses. First, we analyzed trends in the number of nonoral hormonal contraceptives filled at retail pharmacies. We did this to assess whether declines in use of oral contraceptives were offset by increases in use of nonoral contraceptives. Second, we conducted secondary DID regression analyses where we excluded Iowa and Wisconsin from the comparison group. We did this because these states had a temporary change in abortion policy 1 year after *Dobbs* in 2023 where the state became more restrictive. Specifically, in July 2023, abortion was banned at 6 weeks or later in Iowa but in August 2023 it returned to being banned at 22 weeks or later. In Wisconsin, due to legal uncertainty around a pre-*Roe* 1849 law, clinicians stopped offering abortion completely in August until September 12, 2023, when a ruling determined that the law does not apply to abortion, and clinics resumed offering abortion at less than 22 weeks.

## Results

### Prescription Characteristics of Oral Contraceptive Fills in the US

Between March 2021 and October 2023, 142.8 million prescriptions (mean [SD], 4.3 million [390 905] fills per month) for OCPs and 904 269 (mean [SD], 28 206 [3955] fills per month) for ECs were filled at retail pharmacies in the US (Table 1). OCPs were more frequently prescribed by obstetricians and gynecologists (50 255 577 prescriptions [35.2%]) and paid for by commercial insurance (109 595 357 prescriptions [79.6%]). In contrast, ECs were commonly prescribed by nurse practitioners (404 327 prescriptions [44.7%]), with nearly half paid for by Medicaid (425 901 prescriptions [47.2%]). Less than 2% of oral contraceptive fills were prescribed by pharmacists. Levonorgestrel accounted for 472 782 prescription fills for ECs (52.4%) and was more commonly prescribed by pharmacists (13 468 prescriptions [2.84%]) and paid for by Medicaid (280 919 prescriptions [59.2%]) than ulipristal.

**Table 1.** Characteristics of Prescription Fills for Oral Contraceptive Pills and Emergency Contraceptives by Type in the US, March 2021 to October 2023^a^

Characteristic	Prescriptions, No. (%)			
	Oral contraceptive pills (n = 142 824 605)	Emergency contraceptives		
		Overall (n = 904 269)	Levonorgestrel (n = 472 782)	Ulipristal (n = 429 487)
Pharmacy type				
Retail	137 663 602 (96.4)	903 697 (99.9)	474 496 (99.9)	429 201 (99.9)
Mail order	5 171 103 (3.6)	572 (0.06)	286 (0.06)	286 (0.07)
Prescriber specialty				
Obstetrics/gynecology	50 255 577 (35.2)	87 302 (9.7)	50 621 (10.7)	36 681 (8.5)
Primary care^b^	43 634 432 (32.0)	289 759 (32.0)	135 996 (28.6)	153 763 (35.8)
Nurse practitioner	43 634 432 (29.9)	404 327 (44.7)	216 603 (45.6)	187 724 (43.7)
All other	3 824 188 (2.7)	77 209 (8.5)	54 324 (11.4)	22 885 (5.3)
Emergency medicine	301 292 (0.21)	32 081 (3.6)	3 770 (0.79)	28 311 (6.6)
Pharmacists	75 192 (0.05)	13 591 (1.5)	13 468 (2.84)	123 (0.03)
Method of payment				
Cash	3 092 375 (2.3)	20 717 (2.3)	9433 (2.0)	11 284 (2.6)
Commercial	109 595 357 (79.6)	451 820 (50.0)	182 147 (38.4)	269 673 (62.9)
Medicaid^c^	23 362 313 (17.0)	425 901 (47.2)	280 919 (59.2)	144 982 (33.8)

^a^ Source: IQVIA National Prescription Audit and IQVIA PayerTrack, March 2021 to October 2023.

^b^ Includes family practice, osteopathic medicine, internal medicine, pediatrics, general practice, internal medicine or pediatrics, geriatrics, general preventive medicine, and physician assistants.

^c^ Includes fee-for-service and managed Medicaid.

### Trends in Oral Contraceptive Fills by State Abortion Policy in the US

**Figure 1.**
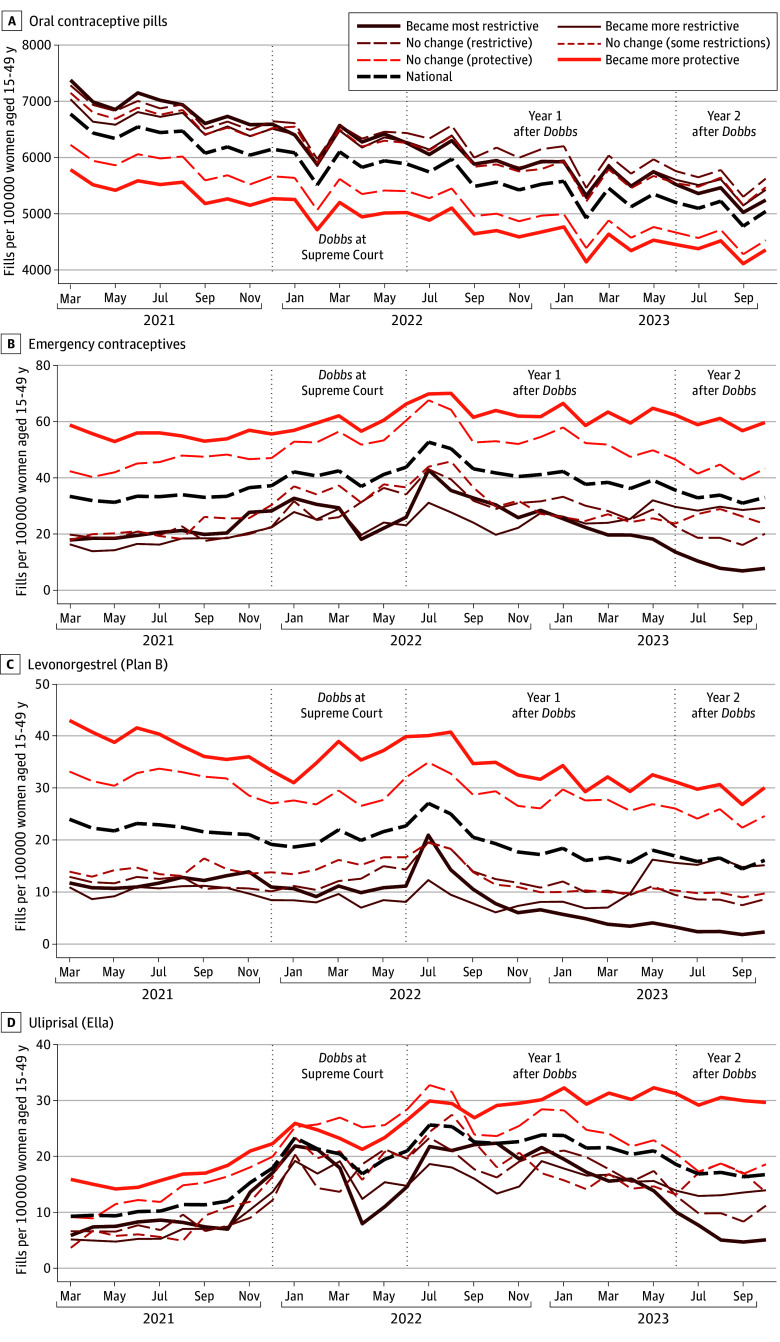
Trends in Oral Contraceptive and Emergency Contraceptive Prescriptions Filled per 100 000 Women of Reproductive Age, 15 to 49 Years, by State Abortion Policy Categories Before and After *Dobbs v Jackson Women’s Health Organization*

Figure 1 depicts trends in monthly fill rates for prescriptions dispensed for OCPs and ECs per 100 000 women of reproductive age in the US and stratified by state abortion policy categories. Nationally, the monthly fill rate for OCPs gradually and significantly declined by 25.6%, from 6784 to 5049 fills per 100 000 women, between March 2021 and October 2023 (*P *for trend < .001). Declines were observed across all 6 state categories. These findings persisted after adjusting for days’ supply for OCPs (eFigure 2 in Supplement 1).

Nationally, monthly fill rates for ECs increased during the period *Dobbs* was being reviewed in the Supreme Court (December 2021 to June 2022) when compared with the pre-*Dobbs* period (March 2021 to November 2021), from 33.3 to 40.5 fills per 100 000 women, respectively (*P *for trend < .001) (Figure 1). EC fills peaked at 52.6 fills per 100 000 women in July 2022, directly following the *Dobbs* decision, and then steadily declined to pre-*Dobbs* levels (32.9 fills per 100 000) by October 2023. In the most restrictive states, fills for ECs ultimately fell below the pre-*Dobbs* level. Between March 2021 and October 2023, the total number of prescriptions for nonoral hormonal contraceptives filled at retail pharmacies steadily declined in the US (eFigure 3 in Supplement 1), including in states that became the most restrictive after *Dobbs* (eFigure 4 in Supplement 1).

### Association Between *Dobbs* and Oral Contraceptive Fills in the Most Restrictive States

**Figure 2.**
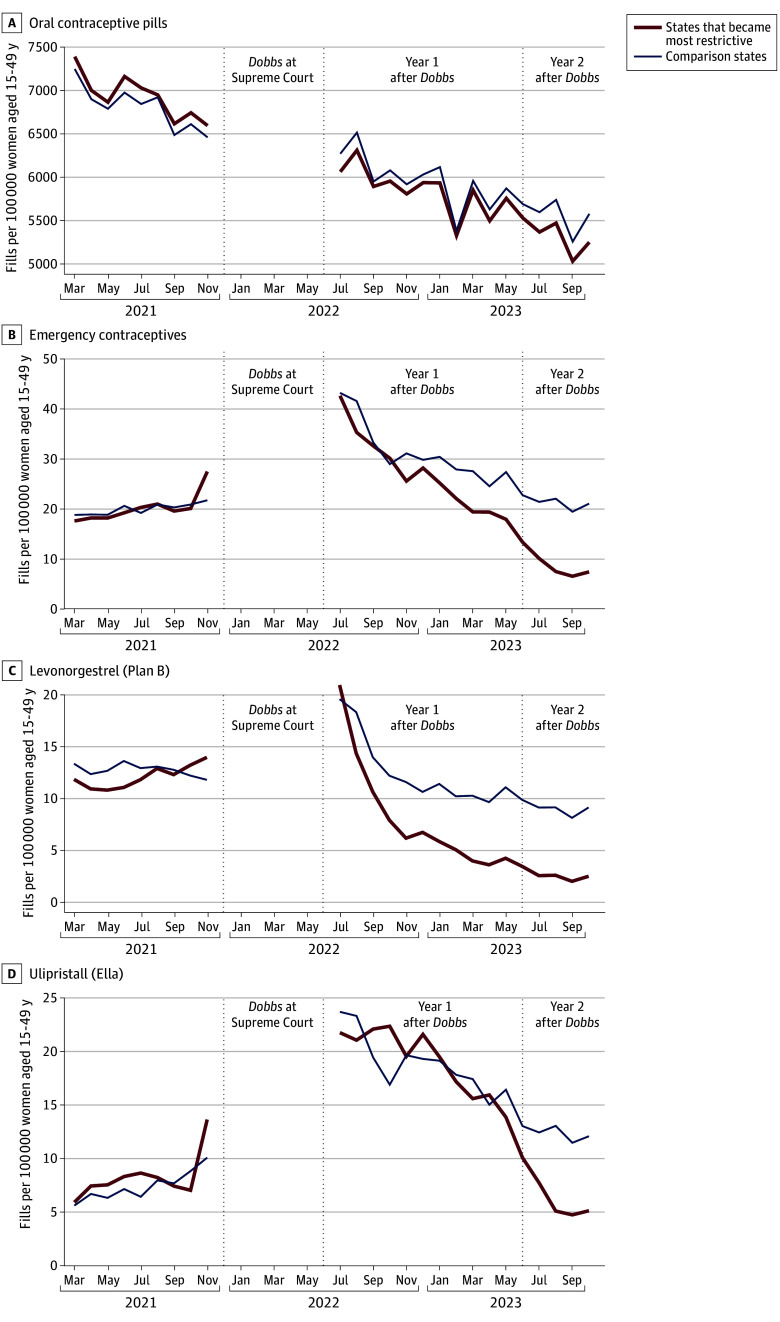
Trends in Oral Contraceptive and Emergency Contraceptive Prescriptions Filled in States That Became Most Restrictive After *Dobbs v Jackson Women’s Health Organization *vs Comparison States, March 2021 to October 2023

In the period before the *Dobbs* oral arguments (from March 2021 to November 2021), trends in monthly fill rates for the primary and secondary outcomes were similar between states that subsequently became most restrictive and comparison states (Figure 2). In the difference-in-differences analysis, the *Dobbs* decision was associated with declines in OCP fills in states that became the most restrictive (4.1% decline with 285.9 fewer fills per 100 000 women; 95% CI, −495.8 to −6.8; *P* = .04) (Table 2). When separately analyzing data from less than 1 year vs 1 year after *Dobbs,* however, declines in OCP fills were greater and statistically significant 1 year after *Dobbs* (July 2023 to October 2023), when the most restrictive states experienced an additional 5.6% decline with 386.0 fewer OCP fills per 100 000 women (95% CI, −676.3 to −63.2; *P* = .02) compared with comparison states.

**Table 2.** Changes in Oral Contraceptive and Emergency Contraceptive Fills Associated With the *Dobbs v Jackson Women’s Health Organization* Decision in the Most Restrictive States, March 2021 to October 2023^a^

Type of contraceptive and time period	Monthly fills per 100 000 women of reproductive age, mean		Regression analysis		*P* value
	Most restrictive states^b^	Comparison states^c^	Difference-in-differences estimate for monthly fills per 100 000 women (95% CI)	% Change^d^	
Oral contraceptive pills					
Pre-*Dobbs* (March 2021-November 2021)	6927.4	6803.6	1 [Reference]	NA	NA
Post-*Dobbs* (July 2022-October 2023)	5687.8	5849.8	−285.9 (−495.8 to −6.8)	−4.13	.04
July 2022-June 2023	5823.1	5951.8	−252.5 (−453.0 to 23.4)	−3.64	.07
July 2023-October 2023	5281.8	5544.0	−386.0 (−676.3 to −63.2)	−5.57	.02
Emergency contraceptives					
Pre-*Dobbs* (March 2021-November 2021)	20.3	20.2	1 [Reference]	NA	NA
Post-*Dobbs* (July 2022-October 2023)	21.6	28.4	−7.0 (−18.2 to 2.2)	−34.2	.11
July 2022-June 2023	26.1	30.8	−4.9 (−16.7 to 6.7)	−24.0	.34
July 2023-October 2023	8.1	21.1	−13.2 (−27.2 to −4.1)	−65.0	.01
Levonorgestrel					
Pre-*Dobbs* (March 2021-November 2021)	12.1	12.8	1 [Reference]	NA	NA
Post-*Dobbs* (July 2022-October 2023)	6.4	11.5	−4.5 (−9.0 to −1.1)	−36.8	.01
July 2022-June 2023	7.8	12.4	−4.0 (−8.9 to −0.4)	−33.0	.03
July 2023-October 2023	2.4	8.9	−5.8 (−11.1 to −2.1)	−48.0	.01
Ulipristal					
Pre-*Dobbs* (March 2021-November 2021)	8.2	7.4	1 [Reference]	NA	NA
Post-*Dobbs* (July 2022-October 2023)	15.2	16.9	−2.5 (−12.4 to 6.4)	−30.5	.60
July 2022-June 2023	18.4	18.4	−0.9 (−10.4 to 10.9)	−10.7	.87
July 2023-October 2023	5.7	12.3	−7.4 (−19.3 to 0.4)	−89.9	.06

Abbreviation: NA, not applicable.

^a^ Source: IQVIA National Prescription Audit (NPA) and IQVIA PayerTrak, March 2021 to October 2023.

^b^ States that became most restrictive: Alabama, Arkansas, Idaho, Kentucky, Louisiana, Mississippi, Missouri, Oklahoma, South Dakota, Tennessee, Texas, and West Virginia.

^c^ Comparison states (medium restrictiveness): Delaware, Florida, Iowa, Kansas, Michigan, Montana, Nevada, New Hampshire, Pennsylvania, Rhode Island, Utah, Virginia, Wisconsin, and Wyoming.

^d^ Percentage change is calculated as (difference-in-difference estimate/pre-*Dobbs* mean × 100).

In contrast, declines in fills for ECs were not significant when data from the full post-*Dobbs* period were pooled together (with 7.0 fewer fills per 100 000 women; 95% CI, −18.2 to 2.2; *P* = .11). During the first year after *Dobbs*, EC fills increased in both groups of states, and a difference-in-differences analysis found no significant additional change in the most restrictive states. Subsequently, 1 year after *Dobbs,* the most restrictive states experienced an additional 65% decrease in EC fills with 13.2 fewer fills per 100 000 women (95% CI, −27.2 to −4.1; *P* = .01).

Increases in EC fills during the first year after *Dobbs* were primarily due to increases in ulipristal fills which offset declines in levonorgestrel fills in the most restrictive states. However, 1 year after *Dobbs,* fills for levonorgestrel and ulipristal declined in the most restrictive states by an additional 48% (5.8 fewer fills per 100 000 women; 95% CI, −11.1 to −2.1) and 89.9% (7.4 fewer fills per 100 000 women; 95% CI, −19.3 to 0.4), respectively. Our findings persisted in sensitivity analyses that excluded Wisconsin and Iowa from the group of comparison states (eFigure 5 and eTable 4 in Supplement 1).

### Changes in Oral Contraceptive Fills in Specific States That Became Most Restrictive After *Dobbs*

**Figure 3.**
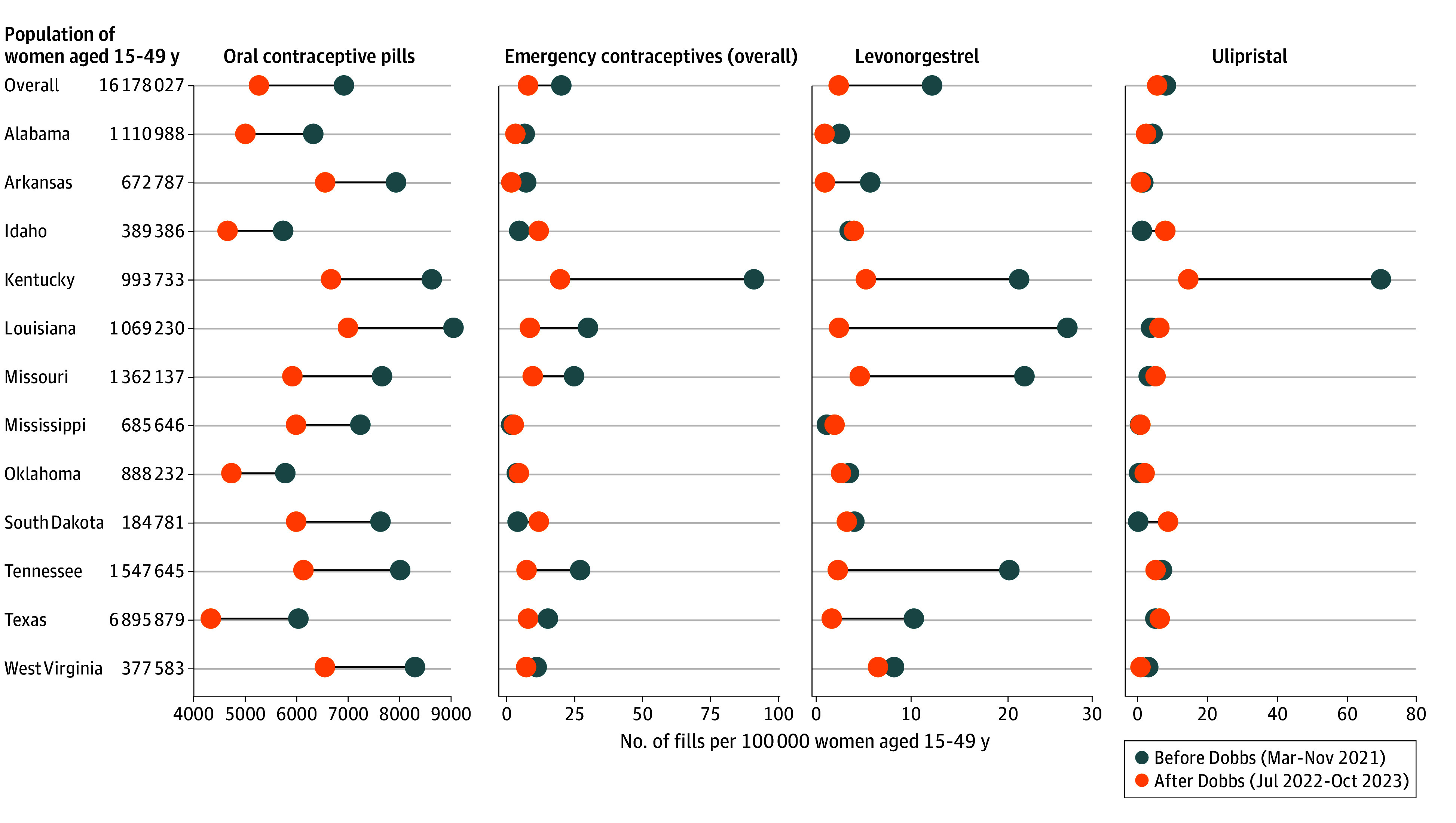
Changes in Prescriptions Filled for Oral Contraceptive Pills and Emergency Contraceptives per 100 000 Women Aged 15 to 49 Years Among States That Became Most Restrictive After the *Dobbs v Jackson Women’s Health Organization *Decision, March 2021 to October 2023 Source: IQVIA National Prescription Audit and IQVIA PayerTrak, March 2021 to October 2023.

Figure 3 summarizes changes in oral contraceptive fills after the *Dobb*s decision in the most restrictive states. When compared with the pre-*Dobbs* period (March 2021 to November 2021), significant declines in monthly fills for OCPs were observed 1 year post-*Dobbs* in all 12 states that became the most restrictive and were most pronounced in Texas, which declined by 28% (from 6046.4 to 4350.5 fills per 100 000 women; *P* < .001). Monthly fills for ECs were lowest in Mississippi before *Dobbs* and decreased 1 year after *Dobbs* in most of the most restrictive states, including Texas. Increases, however, were observed in Idaho (from 4.9 to 12.0 fills per 100 000 women; *P* < .001) and South Dakota (from 4.3 to 12.0 fills per 100 000; *P* < .001) and were due to substantial increases in ulipristal fills post-*Dobbs.*

## Discussion

To our knowledge, this is the first study to evaluate the association between the *Dobbs* decision and use of oral and emergency contraceptives in the US. Using monthly data on dispensed prescriptions from March 2021 through October 2023, we found that *Dobbs* was associated with declines in the use of OCPs in the most restrictive states with a full ban on abortion. We also found that *Dobbs* was associated with immediate, yet transient, increases in ECs followed by substantial declines 1 year after Dobbs in the most restrictive states. These findings are important given ongoing debates regarding protecting contraceptive access post-*Dobbs* in the US^28,29,30^ and the critical role that oral contraceptives play in preventing pregnancy and the need for abortion, particularly in states where access to abortion is strongly restricted.

Consistent with prior studies evaluating the association between implementation of restrictive federal and state abortion policies and access to contraceptives,^20,21,22,23,24^ the declines in fills for OCPs and ECs we document in the most restrictive states after *Dobbs* may be related to the closure of family planning clinics.^25,26^ Approximately 11% of US women of reproductive age rely on publicly-supported family planning clinics for their contraception^10^ and one-third of these clinics provide prescriptions for OCPs or ECs to be filled at outside pharmacies.^27^ In fact, we found that post-*Dobbs* declines in ECs were greatest in the most restrictive states that had closed a larger share of their family-planning clinics. Therefore, *Dobbs* may have worsened access to contraceptives among women who relied on family planning clinics that closed after *Dobbs*.

Confusion about the legality of ECs after *Dobbs*^17^ may have also contributed to declines in fills for ECs, particularly in the most restrictive states where nearly half of women believe levonorgestrel is illegal in their state.^18^ Similar to reports of increases in purchases and stockpiling of OTC levonorgestrel during the weeks directly before and after *Dobbs*,^31^ we found immediate, yet transient, increases in prescription fills for levonorgestrel that were followed by substantial declines. Although declines in levonorgestrel fills during the first year of *Dobbs* were offset by increases in ulipristal, ulipristal subsequently declined 1 year after *Dobbs*, contributing to large decreases in fills for ECs in the most restrictive states. Future research can assess whether efforts to clarify the legality of ECs and address misinformation defining them as abortifacients^11^ could improve access to ECs among women who prefer to use them.

Restrictive contraceptive policies, including the exclusion of ECs from contraceptive coverage mandates,^8,9,10^ the lack of Medicaid coverage for over-the-counter ECs,^31^ and policies that allow pharmacists to refuse to dispense contraceptives due to moral, ethical, or religious objections,^11,12^ may also contribute to lower rates of use in the most restrictive states. For example, we found that fills for ECs before *Dobbs* were lowest in Mississippi, which is the only state that prohibits Medicaid coverage for levonorgestrel.^32^ Conversely, EC fills after *Dobbs* increased the most in Idaho, which, among states with the most restrictive abortion policies, is the only one that allows pharmacists to prescribe ECs to women without any age restrictions.^12^ Future research should investigate the impact of policies that expand pharmacist ability to prescribe ECs in states like Idaho that also have restrictive abortion policies.

The declines in the use of oral contraceptives we observe after *Dobbs* may contribute to changes in overall use of contraception, a key tool in the prevention of pregnancy in states where abortion access has been severely restricted. A recent study^33^ of 4 states with less restrictive abortion policies found *Dobbs* was associated with declines in contraceptive visits and increases in the use of condoms. In contrast to several reports suggesting *Dobbs* may have increased demand for LARCs, including intrauterine devices (IUDs), and permanent contraception,^34^ this study found no change in the use of IUDs or permanent contraception.^33^ Our sensitivity analyses found that prescriptions for nonoral hormonal contraceptives filled at pharmacies (eg, contraceptive patch or vaginal ring) did not increase after *Dobbs,* including in the most restrictive states, suggesting the declines in oral contraceptive fills we documented were not offset by increases in the use of nonoral hormonal contraceptives. Future work should examine how overall use of contraception, including permanent and reversible methods and that are not self-administered, changed post-*Dobbs* in states with the most restrictive abortion policies.

### Limitations

Our analyses had several limitations. First, our data are limited to prescription medications filled at retail or mail-order pharmacies and do not capture over-the-counter sales. However, a prior study^23^ found that the closure of abortion and family planning clinics in Texas was not associated with changes in OTC purchases of ECs. Therefore, increased demand of OTC levonorgestrel during the weeks directly preceding and following Dobbs was likely short-lived, similar to our findings on prescription fills for levonorgestrel. Second, we used aggregate, not individual-level, data on prescriptions filled. Nonetheless, our finding that 11.5% of women using OCPs filled at retail pharmacies is consistent with national, self-reported individual-level, survey-based estimates (see eTable 1 in Supplement 1).^35^ Third, our data may not capture oral contraceptives prescribed and mailed through online platforms; however, such channels account for fewer than 5% of all prescriptions for contraceptives in the US.^10^ Fourth, changes in the use of oral contraceptives after *Dobbs* may be intertwined with dynamic federal and state policies governing both abortion and contraceptive coverage, access, and affordability, that may influence our findings. However, the parallel trends between the most restrictive and comparison states observed before *Dobbs* are reassuring. Additionally, our study design compared changes before vs after *Dobbs* and therefore could not be used to assess the impact of policy changes that reduce abortion restrictions. Unlike the states that enacted full bans on abortion within 2 months after *Dobbs*, the states that made their policy more protective of abortion access typically made these changes 6 to 8 months after *Dobbs* and legally could have made these changes before *Dobbs*.

## Conclusions

In this cohort study of prescriptions filled at US pharmacies, the *Dobbs* decision was associated with declines in fills for oral contraceptives, particularly ECs, in states that implemented the most restrictive abortion policies. Given the role of daily OCPs and ECs in preventing pregnancy and the need for an abortion, efforts to improve and protect access to oral contraceptives are needed, especially for ECs in states where abortion is most strongly restricted.

## Notes

Supplement 1. **eFigure 1.** Trends in Prescriptions Fills for Oral Hormonal Contraceptives at Retail Pharmacies in the US **eTable 1. **Proportion of Women That Fill Their Oral Contraceptive Pills at Retail Pharmacies in the US **eTable 2. **Changes in State Abortion Policies After the US Supreme Court’s *Dobbs* Decision, From June 2022 to October 2023 **eTable 3. **States by Abortion Policy Category, From June 2022 to October 2023 **eFigure 2. **Trends in Oral Contraceptive Fills per 100 000 Women of Reproductive Age, Nationally and by State Abortion Policy Category, March 2021 to October 2023 **eFigure 3. **Trends in the Number of Nonoral Hormonal Contraceptive Prescriptions Filled at Retail Pharmacies in the US **eFigure 4. **Trends in the Number of Nonoral Hormonal Contraceptives Filled at Retail Pharmacies in the States With the Most Restrictive Abortion Policy, March 2021 to October 2023 **eFigure 5. **Trends in Fills for Oral Contraceptives per 100 000 Women of Reproductive Age, in States Whose Abortion Policy Became Most Restrictive vs Comparison States (Excluding Iowa and Wisconsin), March 2021 to October 2023 **eTable 4. **Changes in Monthly Fills for Oral Contraceptives per 100 000 Women of reproductive Age in States Whose Abortion Policy Became Most Restrictive vs Comparison States (Excluding Iowa and Wisconsin), March 2021 to October 2023

Supplement 2.
Data Sharing Statement
