# Errors in Introduction, Results, and Discussion

**DOI:** 10.1001/jamanetworkopen.2024.29077

**Published:** 2024-07-25

In the Original Investigation titled “Use of Oral and Emergency Contraceptives After the US Supreme Court’s *Dobbs* Decision,”^1^ published June 26, 2024, there were errors in the Introduction, Results, and Discussion. In addition to typographical errors, the article stated there were 285.0 fewer oral contraceptive prescription fills per 100 000 women, and it should have stated there were 285.9 fewer prescription fills. This article has been corrected.^1^
