# Supplementary material for: Use of Oral and Emergency Contraceptives After the US Supreme Court’s Dobbs Decision
Source: JAMA Netw Open. 2024 Jun 26;7(6):e2418620. doi: 10.1001/jamanetworkopen.2024.18620 (PMC11208973; doi:10.1001/jamanetworkopen.2024.18620)
Supplement: Supplement 1. — eFigure 1. Trends in Prescriptions Fills for Oral Hormonal Contraceptives at Retail Pharmacies in the US eTable 1. Proportion of Women That Fill Their Oral Contraceptive Pills at Retail Pharmacies in the US eTable 2. Changes in State Abortion Policies After the US Supreme Court’s Dobbs Decision, From June 2022 to October 2023 eTable 3. States by Abortion Policy Category, From June 2022 to October 2023 eFigure 2. Trends in Oral Contraceptive Fills per 100 000 Women of Reproductive Age, Nationally and by State Abortion Policy Category, March 2021 to October 2023 eFigure 3. Trends in the Number of Nonoral Hormonal Contraceptive Prescriptions Filled at Retail Pharmacies in the US eFigure 4. Trends in the Number of Nonoral Hormonal Contraceptives Filled at Retail Pharmacies in the States With the Most Restrictive Abortion Policy, March 2021 to October 2023 eFigure 5. Trends in Fills for Oral Contraceptives per 100 000 Women of Reproductive Age, in States Whose Abortion Policy Became Most Restrictive vs Comparison States (Excluding Iowa and Wisconsin), March 2021 to October 2023 eTable 4. Changes in Monthly Fills for Oral Contraceptives per 100 000 Women of reproductive Age in States Whose Abortion Policy Became Most Restrictive vs Comparison States (Excluding Iowa and Wisconsin), March 2021 to October 2023 [file jamanetwopen-e2418620-s001.pdf]

Supplemental Online Content

Qato DM, Myerson R,Shooshtari A, Guadamuz JS, Alexander GC. Changes in use of oral and emergency contraceptives after the US Supreme Court's Dobbs decision. *JAMA Netw Open*. 2024;7(6):e2418620. doi:10.1001/jamanetworkopen.2024.18620

eFigure 1. Trends in Prescriptions Fills for Oral Hormonal Contraceptives at Retail Pharmacies in the US
eTable 1. Proportion of Women Who Fill Their Oral Contraceptive Pills at Retail Pharmacies in the US
eTable 2. Changes in State Abortion Policies after the U.S. Supreme Court's *Dobbs* decision, from June 2022 to October 2023
eTable 3. States by Abortion Policy Category, from June 2022 to October 2023
eFigure 2. Trends in Oral Contraceptive Fills per 100 000 Women of Reproductive Age, Nationally and by State Abortion Policy Category, March 2021 to October 2023
eFigure 3. Trends in the Number of Nonoral Hormonal Contraceptives Filled at Retail Pharmacies In the US
eFigure 4. Trends in the Number of Nonoral Hormonal Contraceptives Filled at Retail Pharmacies in the States With the Most Restrictive Abortion Policy, March 2021 to October 2023
eFigure 5. Trends in Fills for Oral Contraceptives per 100 000 Women of Reproductive Age, in States Whose Abortion Policy Became Most Restrictive vs Comparison States (Excluding Iowa and Wisconsin), March 2021 to October 2023
eTable 4. Changes in Monthly Fills for Oral Contraceptives Per 100 000 Women of Reproductive Age in States Whose Abortion Policy Became Most Restrictive vs Comparison States (Excluding Iowa and Wisconsin), March 2021 to October 2023

This supplemental material has been provided by the authors to give readers additional information about their work.

© 2024 Qato DM et al. *JAMA Network Open*.

eFigure 1. Trends in Prescriptions Fills for Oral Hormonal Contraceptives at Retail Pharmacies in the US

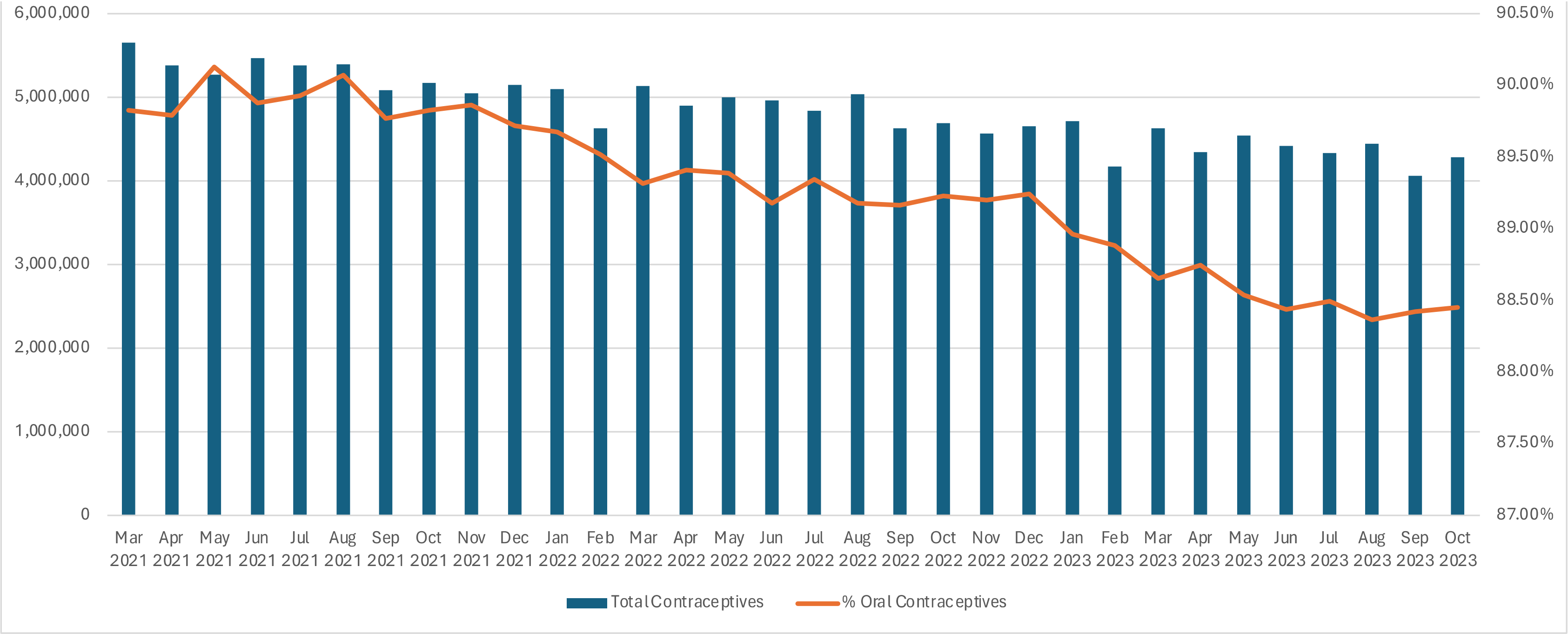

Source: IQVIA National Prescription Audit® and IQVIA PayerTrak™, March 2021-October 2023

© 2024 Qato DM et al. *JAMA Network Open*.

eTable 1. Proportion of Women Who Fill Their Oral Contraceptive Pills at Retail Pharmacies in the US

Population of US Women aged 15-49 years in the U.S.	74,525,181
Total Number of Prescriptions Filled for OCPs at Retail Pharmacies in 2019 (based on IQVIA NPA)	65,0777,772
Total Number of Prescriptions Filled for OCPs at Retail Pharmacies for a 12-month supply in 2019 (Based on IQVIA NPA)	8,850,953
**Estimated Percentage. of US Women aged 15-49 years that regularly used OCPs in 2019**	**11.5%**

According to the NCHS estimates for 2019[1], 14% of women aged 15-49 years used OCPs. Using IQVIA data for the same year, there were a total of 65,0777,772 prescriptions filled for OCPs in 2019 and 8,850,953 OCPs prescriptions adjusted for 336 days in the year (28-day supply every month for 12-months). Therefore, given 74,525,181 women of reproductive age 15-49 years, in 2019 ~ 11.8% (8,850,953/74,525,181) used OCPs regularly. Given individual-level NCHS estimates of 14%, these findings indicate that ~85% of women that regularly use OCPs filled them at retail pharmacies.

1. Daniels K, Abma JC. Current contraceptive status among women aged 15–49: United States, 2017–2019. NCHS Data Brief, no 388. Hyattsville, MD: National Center for Health Statistics. 2020. https://www.cdc.gov/nchs/data/databriefs/db388-H.pdf

© 2024 Qato DM et al. *JAMA Network Open*.

eTable 2. Changes in State Abortion Policies after Dobbs, from June 2022 to October 2023

State Full Name	State Category	Jun-22	Jul-22	Aug-22	Sep-22	Oct-22	Nov-22	Dec-22	Jan-23	Feb-23	Mar-23	Apr-23	May-23	Jun-23	Jul-23	Aug-23	Sep-23	Oct-23
Maryland	Became more protective	Some restrictions,	Protective	Protective	Protective	Protective	Protective	Protective	Protective	Protective	Protective	Protective	Protective	Protective	Protective	Protective	Protective	Protective
California	Became more protective	Protective	Protective	Protective	Protective	Protective	Very Protective	Very Protective	Very Protecti	Very Prote	Very Protective	Very Prote	Very Prote	Very Protectiv	Very Protective	Very Protecti	Very Protective	Very Protective
New Jersey	Became more protective	Protective	Protective	Protective	Protective	Protective	Protective	Protective	Very Protecti	Very Prote	Very Protective	Very Prote	Very Prote	Very Protectiv	Very Protective	Very Protecti	Very Protective	Very Protective
New Mexico	Became more protective	Protective	Protective	Protective	Protective	Protective	Protective	Protective	Very Protecti	Very Prote	Very Protective	Very Prote	Very Prote	Very Protectiv	Very Protective	Very Protecti	Very Protective	Very Protective
New York	Became more protective	Protective	Protective	Protective	Protective	Protective	Protective	Protective	Very Protecti	Very Prote	Very Protective	Very Prote	Very Prote	Very Protectiv	Very Protective	Very Protecti	Very Protective	Very Protective
Vermont	Became more protective	Protective	Protective	Protective	Protective	Protective	Very Protective	Very Protective	Very Protecti	Very Prote	Very Protective	Very Prote	Very Prote	Very Protectiv	Very Protective	Very Protecti	Very Protective	Very Protective
Massachusetts	Became more protective	Some restrictions,	Some restrictio	Protective	Protective	Protective	Protective	Protective	Protective	Protective	Protective	Protective	Protective	Protective	Protective	Protective	Protective	Protective
Connecticut	Became more protective	Some restrictions,	Some restrictio	Some restrictior	Some restrictio	Some restrictio	Some restrictic	Some restrictions	Protective	Protective	Protective	Protective	Protective	Protective	Protective	Protective	Protective	Protective
District of Columbi	Became more protective	Some restrictions,	Some restrictio	Some restrictior	Some restrictio	Some restrictio	Protective	Protective	Protective	Protective	Protective	Protective	Protective	Protective	Protective	Protective	Protective	Protective
Hawaii	Became more protective	Some restrictions,	Some restrictio	Some restrictior	Some restrictio	Some restrictio	Some restrictic	Some restrictions	Protective	Protective	Protective	Protective	Protective	Protective	Protective	Protective	Protective	Protective
Minnesota	Became more protective	Some restrictions,	Some restrictio	Some restrictior	Some restrictio	Some restrictio	Some restrictic	Some restrictions	Some restrict	Some resti	Some restriction:	Protective	Protective	Protective	Protective	Very Protecti	Very Protective	Very Protective
South Carolina	Became more restrictive	Very restrictive	Very Restrictive	Restrictive	Restrictive	Restrictive	Restrictive	Restrictive	Restrictive	Restrictive	Restrictive	Restrictive	Restrictive	Restrictive	Restrictive	Most Restric	Most Restrictive	Most Restrictive
North Dakota	Became more restrictive	Restrictive	Restrictive	Restrictive	Restrictive	Restrictive	Restrictive	Restrictive	Restrictive	Restrictive	Restrictive	Most Resti	Most Resti	Most Restricti	Most Restrictive	Most Restric	Most Restrictive	Most Restrictive
Indiana	Became more restrictive	Restrictive	Restrictive	Most Restrictive	Restrictive	Restrictive	Restrictive	Restrictive	Restrictive	Restrictive	Restrictive	Restrictive	Restrictive	Restrictive	Restrictive	Most Restric	Most Restrictive	Most Restrictive
North Carolina	Became more restrictive	Some restrictions,	Some restrictio	Restrictive	Restrictive	Restrictive	Restrictive	Restrictive	Restrictive	Restrictive	Restrictive	Restrictive	Restrictive	Restrictive	Very Restrictive	Very Restrict	Very Restrictive	Very Restrictive
Georgia	Became more restrictive	Restrictive	Very Restrictive	Very Restrictive	Very Restrictive	Very Restrictive	Very Restrictiv	Very Restrictive	Very Restricti	Very Restri	Very Restrictive	Very Restri	Very Restri	Very Restricti	Very Restrictive	Very Restrict	Very Restrictive	Very Restrictive
Nebraska	Became more restrictive	Restrictive	Restrictive	Restrictive	Restrictive	Restrictive	Restrictive	Restrictive	Restrictive	Restrictive	Restrictive	Restrictive	Very Restri	Very Restricti	Very Restrictive	Very Restrict	Very Restrictive	Very Restrictive
Alabama	Became most restrictive (by.	Restrictive	Most Restrictive	Most Restrictive	Most Restrictive	Most Restrictive	Most Restricti	Most Restrictive	Most Restrict	Most Resti	Most Restrictive	Most Resti	Most Resti	Most Restricti	Most Restrictive	Most Restric	Most Restrictive	Most Restrictive
Arkansas	Became most restrictive (by.	Most Restrictive	Most Restrictive	Most Restrictive	Most Restrictive	Most Restrictive	Most Restricti	Most Restrictive	Most Restrict	Most Resti	Most Restrictive	Most Resti	Most Resti	Most Restricti	Most Restrictive	Most Restric	Most Restrictive	Most Restrictive
Mississippi	Became most restrictive (by.	Restrictive	Most Restrictive	Most Restrictive	Most Restrictive	Most Restrictive	Most Restricti	Most Restrictive	Most Restrict	Most Resti	Most Restrictive	Most Resti	Most Resti	Most Restricti	Most Restrictive	Most Restric	Most Restrictive	Most Restrictive
Missouri	Became most restrictive (by.	Most Restrictive	Most Restrictive	Most Restrictive	Most Restrictive	Most Restrictive	Most Restricti	Most Restrictive	Most Restrict	Most Resti	Most Restrictive	Most Resti	Most Resti	Most Restricti	Most Restrictive	Most Restric	Most Restrictive	Most Restrictive
Oklahoma	Became most restrictive (by.	Most Restrictive	Most Restrictive	Most Restrictive	Most Restrictive	Most Restrictive	Most Restricti	Most Restrictive	Most Restrict	Most Resti	Most Restrictive	Most Resti	Most Resti	Most Restricti	Most Restrictive	Most Restric	Most Restrictive	Most Restrictive
South Dakota	Became most restrictive (by.	Most Restrictive	Most Restrictive	Most Restrictive	Most Restrictive	Most Restrictive	Most Restricti	Most Restrictive	Most Restrict	Most Resti	Most Restrictive	Most Resti	Most Resti	Most Restricti	Most Restrictive	Most Restric	Most Restrictive	Most Restrictive
Texas	Became most restrictive (by.	Very restrictive	Most Restrictive	Most Restrictive	Most Restrictive	Most Restrictive	Most Restricti	Most Restrictive	Most Restricti	Most Restri	Most Restrictive	Most Restri	Most Resti	Most Restricti	Most Restrictive	Most Restric	Most Restrictive	Most Restrictive
Idaho	Became most restrictive (by.	Restrictive	Restrictive	Most Restrictive	Most Restrictive	Most Restrictive	Most Restricti	Most Restrictive	Most Restrict	Most Resti	Most Restrictive	Most Resti	Most Resti	Most Restricti	Most Restrictive	Most Restric	Most Restrictive	Most Restrictive
Louisiana	Became most restrictive (by.	Restrictive	Restrictive	Most Restrictive	Most Restrictive	Most Restrictive	Most Restricti	Most Restrictive	Most Restrict	Most Resti	Most Restrictive	Most Resti	Most Resti	Most Restricti	Most Restrictive	Most Restric	Most Restrictive	Most Restrictive
West Virginia	Became most restrictive (by.	Restrictive	Restrictive	Most Restrictive	Most Restrictive	Most Restrictive	Most Restricti	Most Restrictive	Most Restrict	Most Resti	Most Restrictive	Most Resti	Most Resti	Most Restricti	Most Restrictive	Most Restric	Most Restrictive	Most Restrictive
Kentucky	Became most restrictive (by.	Restrictive	Very Restrictive	Most Restrictive	Most Restrictive	Most Restrictive	Most Restricti	Most Restrictive	Most Restrict	Most Resti	Most Restrictive	Most Resti	Most Resti	Most Restricti	Most Restrictive	Most Restric	Most Restrictive	Most Restrictive
Tennessee	Became most restrictive (by.	Very restrictive	Very Restrictive	Most Restrictive	Most Restrictive	Most Restrictive	Most Restricti	Most Restrictive	Most Restrict	Most Resti	Most Restrictive	Most Resti	Most Resti	Most Restricti	Most Restrictive	Most Restric	Most Restrictive	Most Restrictive
Oregon	No change - protective	Most protective	Most protective	Most protective	Most protective	Most protective	Most protectiv	Most protective	Most protecti	Most prote	Most protective	Most prote	Most prote	Most protecti	Most Protective	Most Protect	Most Protective	Most Protective
Alaska	No change - protective	Protective	Protective	Protective	Protective	Protective	Protective	Protective	Protective	Protective	Protective	Protective	Protective	Protective	Protective	Protective	Protective	Protective
Colorado	No change - protective	Protective	Protective	Protective	Protective	Protective	Protective	Protective	Protective	Protective	Protective	Protective	Protective	Protective	Protective	Protective	Protective	Protective
Illinois	No change - protective	Protective	Protective	Protective	Protective	Protective	Protective	Protective	Protective	Protective	Protective	Protective	Protective	Protective	Protective	Protective	Protective	Protective
Maine	No change - protective	Protective	Protective	Protective	Protective	Protective	Protective	Protective	Protective	Protective	Protective	Protective	Protective	Protective	Protective	Protective	Protective	Protective
Washington	No change - protective	Protective	Protective	Protective	Protective	Protective	Protective	Protective	Protective	Protective	Protective	Protective	Protective	Protective	Protective	Protective	Protective	Protective
Florida	No change - restrictive	Restrictive	Restrictive	Restrictive	Restrictive	Restrictive	Restrictive	Restrictive	Restrictive	Restrictive	Restrictive	Restrictive	Restrictive	Restrictive	Restrictive	Restrictive	Restrictive	Restrictive
Iowa	No change - restrictive	Restrictive	Restrictive	Restrictive	Restrictive	Restrictive	Restrictive	Restrictive	Restrictive	Restrictive	Restrictive	Restrictive	Restrictive	Restrictive	Very Restrictive	Restrictive	Restrictive	Restrictive
Kansas	No change - restrictive	Restrictive	Restrictive	Restrictive	Restrictive	Restrictive	Restrictive	Restrictive	Restrictive	Restrictive	Restrictive	Restrictive	Restrictive	Restrictive	Restrictive	Restrictive	Restrictive	Restrictive
Pennsylvania	No change - restrictive	Restrictive	Restrictive	Restrictive	Restrictive	Restrictive	Restrictive	Restrictive	Restrictive	Restrictive	Restrictive	Restrictive	Restrictive	Restrictive	Restrictive	Restrictive	Restrictive	Restrictive
Utah	No change - restrictive	Restrictive	Restrictive	Restrictive	Restrictive	Restrictive	Restrictive	Restrictive	Restrictive	Restrictive	Restrictive	Restrictive	Restrictive	Restrictive	Restrictive	Restrictive	Restrictive	Restrictive
Wisconsin	No change - restrictive	Restrictive	Restrictive	Restrictive	Restrictive	Restrictive	Restrictive	Restrictive	Restrictive	Restrictive	Restrictive	Restrictive	Restrictive	Restrictive	Restrictive	Most Restric	Restrictive	Restrictive
Delaware	No change - some restriction	Some restrictions,	Some restrictio	Some restrictior	Some restrictio	Some restrictio	Some restrictic	Some restrictions	Some restrict	Some resti	Some restriction:	Some resti	Some resti	Some restricti	Some restrictio	Some restric	Some restriction	Some restrictions
Michigan	No change - some restriction	Some restrictions,	Some restrictio	Some restrictior	Some restrictio	Some restrictio	Some restrictic	Some restrictions	Some restrict	Some resti	Some restriction:	Some resti	Some resti	Some restricti	Some restrictio	Some restric	Some restriction	Some restrictions
Montana	No change - some restriction	Some restrictions,	Some restrictio	Some restrictior	Some restrictio	Some restrictio	Some restrictic	Some restrictions	Some restrict	Some resti	Some restriction:	Some resti	Some resti	Some restricti	Some restrictio	Some restric	Some restriction	Some restrictions
Nevada	No change - some restriction	Some restrictions,	Some restrictio	Some restrictior	Some restrictio	Some restrictio	Some restrictic	Some restrictions	Some restrict	Some resti	Some restriction:	Some resti	Some resti	Some restricti	Some restrictio	Some restric	Some restriction	Some restrictions
New Hampshire	No change - some restriction	Some restrictions,	Some restrictio	Some restrictior	Some restrictio	Some restrictio	Some restrictic	Some restrictions	Some restricti	Some resti	Some restriction:	Some resti	Some resti	Some restricti	Some restrictio	Some restric	Some restriction	Some restrictions
Rhode Island	No change - some restriction	Some restrictions,	Some restrictio	Some restrictior	Some restrictio	Some restrictio	Some restrictic	Some restrictions	Some restrict	Some resti	Some restriction:	Some resti	Some resti	Some restricti	Some restrictio	Some restric	Some restriction	Some restrictions
Virginia	No change - some restriction	Some restrictions,	Some restrictio	Some restrictior	Some restrictio	Some restrictio	Some restrictic	Some restrictions	Some restrict	Some resti	Some restriction:	Some resti	Some resti	Some restricti	Some restrictio	Some restric	Some restriction	Some restrictions
Wyoming	No change - some restriction	Some restrictions,	Some restrictio	Some restrictior	Some restrictio	Some restrictio	Some restrictic	Some restrictions	Some restrict	Some resti	Some restriction:	Some resti	Some resti	Some restricti	Some restrictio	Some restric	Some restriction	Some restrictions
Ohio	Other pattern	Very restrictive	Very Restrictive	Restrictive	Restrictive	Restrictive	Restrictive	Restrictive	Restrictive	Restrictive	Restrictive	Restrictive	Restrictive	Restrictive	Restrictive	Restrictive	Restrictive	Restrictive
Arizona	Other pattern	Restrictive	Restrictive	Restrictive	Most Restrictive	Very Restrictive	Very Restrictiv	Very Restrictive	Very Restricti	Very Restri	Very Restrictive	Very Restri	Very Restri	Very Restricti	Restrictive	Restrictive	Restrictive	Restrictive

Source: State Abortion Categories based on authors' analysis of Guttmacher Institute 'Interactive Map of State Abortion Policies' from June 2022-October 2023

© 2024 Qato DM et al. *JAMA Network Open*.

eTable 3. States by Abortion Policy Category, from June 2021 to October 2023[a]

Abortion Category	State	Population Total
Became more protective	Overall	21,983,830
	MD	1,392,539
	CA	9,325,528
	NJ	1,981,963
	NM	458,985
	NY	4,528,886
	VT	134,194
	MA	1,616,546
	CT	791,887
	DC	208,347
	HI	305,587
	MN	1,239,368
Became more restrictive	Overall	8,176,283
	NC	2,398,192
	GA	2,531,208
	NE	425,952
	SC	1,141,545
	ND	167,627
	IN	1,511,759
Became most restrictive (by August and stayed most restrictive)	Overall	16,178,027
	AL	1,110,988
	AR	672,787
	MS	685,646
	MO	1,362,137
	OK	888,232
	SD	184,781
	TX	6,895,879
	ID	389,386
	LA	1,069,230
	WV	377,583
	KY	993,733
	TN	1,547,645
No change - protective	Overall	7,383,209
	OR	950,105

© 2024 Qato DM et al. *JAMA Network Open*.

	State	Population Total
	AK	166,155
	CO	1,339,182
	IL	2,923,898
	ME	276,560
	WA	1,727,309
No change - restrictive	Overall	10,685,651
	FL	4,540,909
	IA	684,685
	KS	643,059
	PA	2,781,360
	UT	773,607
	WI	1,262,031
No change - some restrictions	Overall	5,943,960
	DE	210,273
	MI	2,194,320
	MT	223,168
	NV	691,806
	NH	289,670
	RI	241,982
	VA	1,968,054
	WY	124,687
Other pattern	Overall	4,174,221
	OH	2,577,743
	AZ	1,596,478

a State abortion categories based on authors' analysis of Guttmacher Institute's 'Interactive Map of State Abortion Policies' from June 2022-October 2023

© 2024 Qato DM et al. *JAMA Network Open*.

eFigure 2. Trends in Oral Contraceptive Fills per 100 000 Women Aged 15 to 49 Years Nationally and by State Category (Adjusting for 28-Day Supply) March 2021 to October 2023

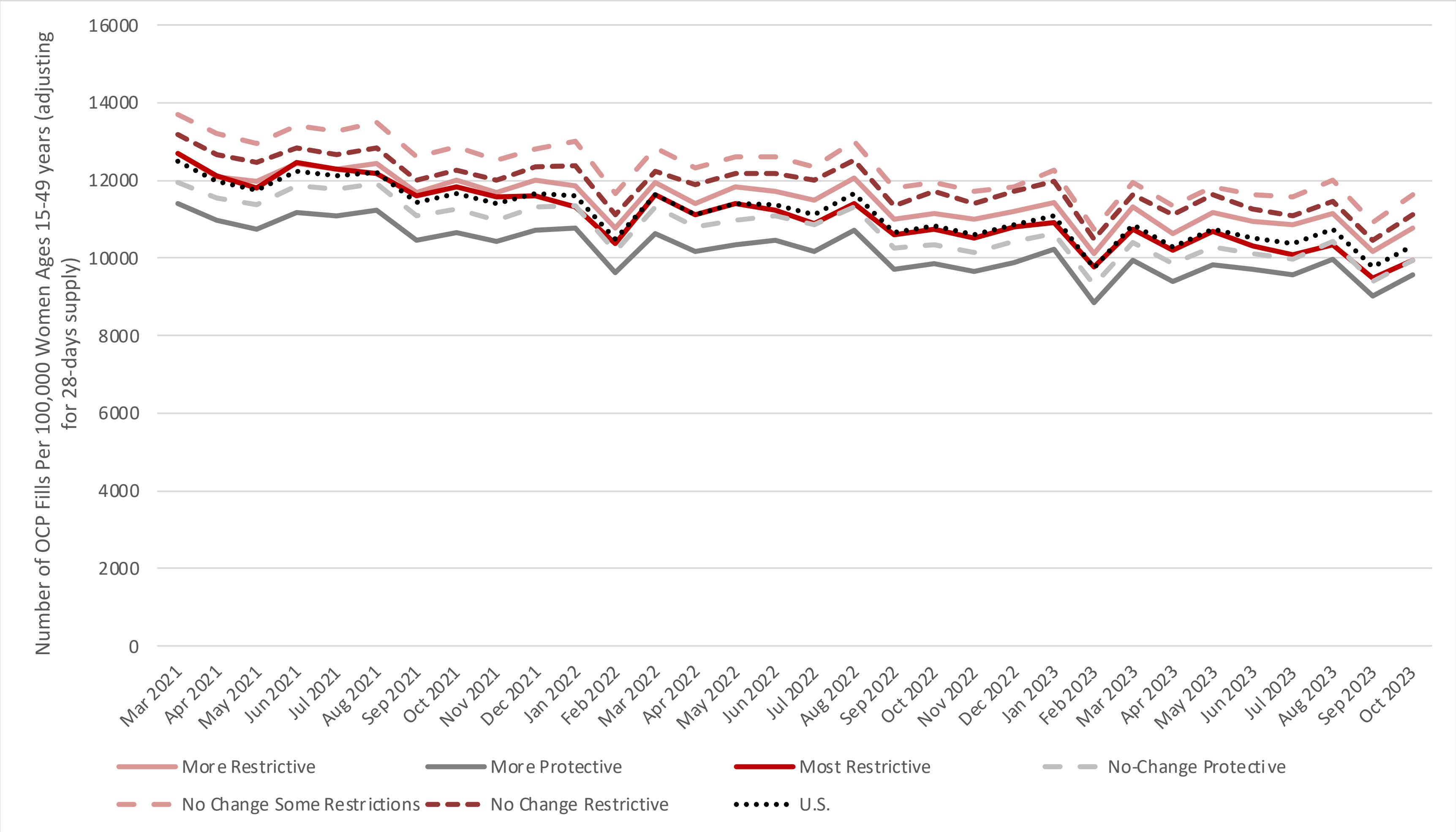

Source: IQVIA National Prescription Audit® and IQVIA PayerTrak™, March 2021-October 2023. Total Number of Tablets for Oral Contraceptives was divided by 28-days to adjust for prescriptions filled for varying days' supply.

© 2024 Qato DM et al. *JAMA Network Open.*

eFigure 3. Trends in the Number of Nonoral Hormonal Contraceptives Filled at Retail Pharmacies In the US

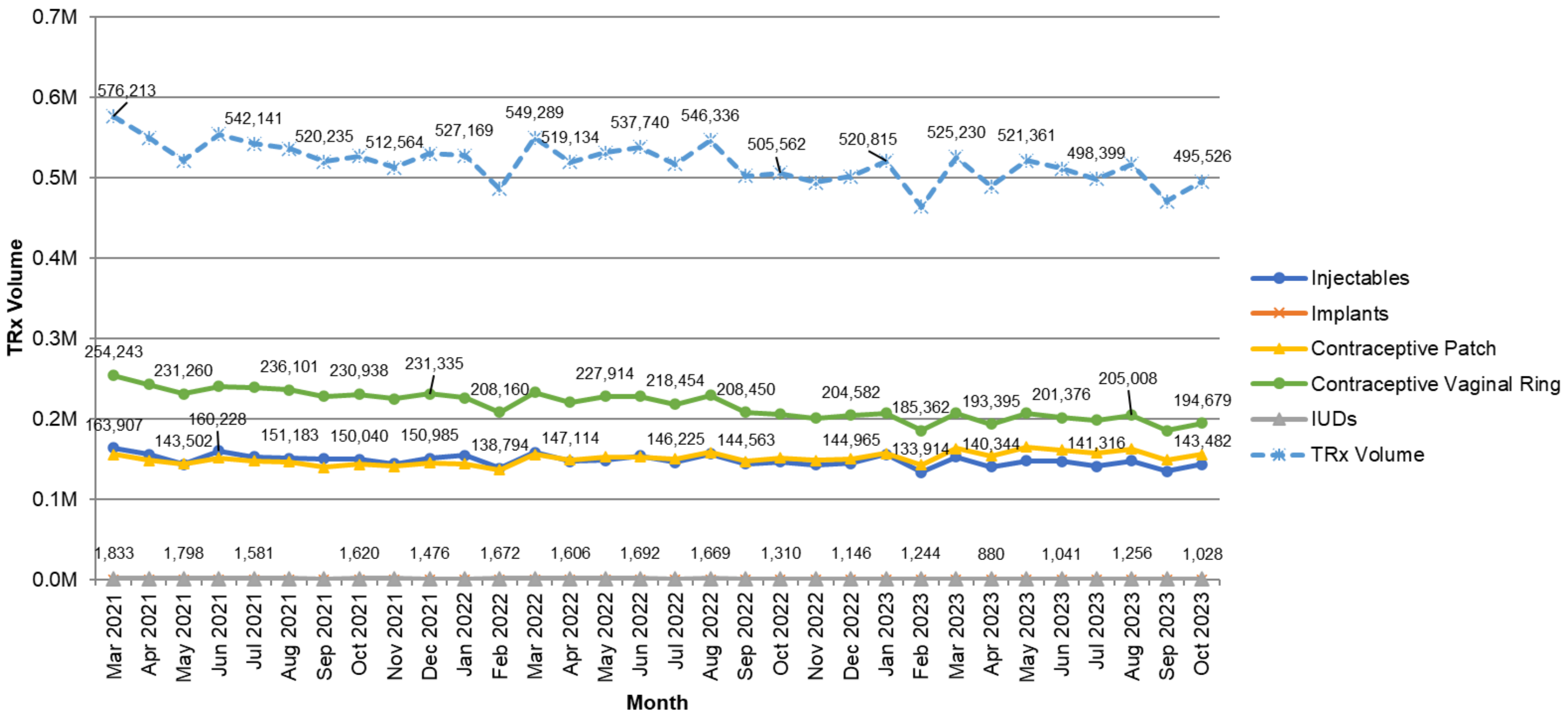

Source: IQVIA National Prescription Audit® PayerTrak™, March 2021-October 2023

© 2024 Qato DM et al. *JAMA Network Open*.

eFigure 4. Trends in the Number of Nonoral Hormonal Contraceptives Filled at Retail Pharmacies in the Most Restrictive States, March 2021 toOctober 2023

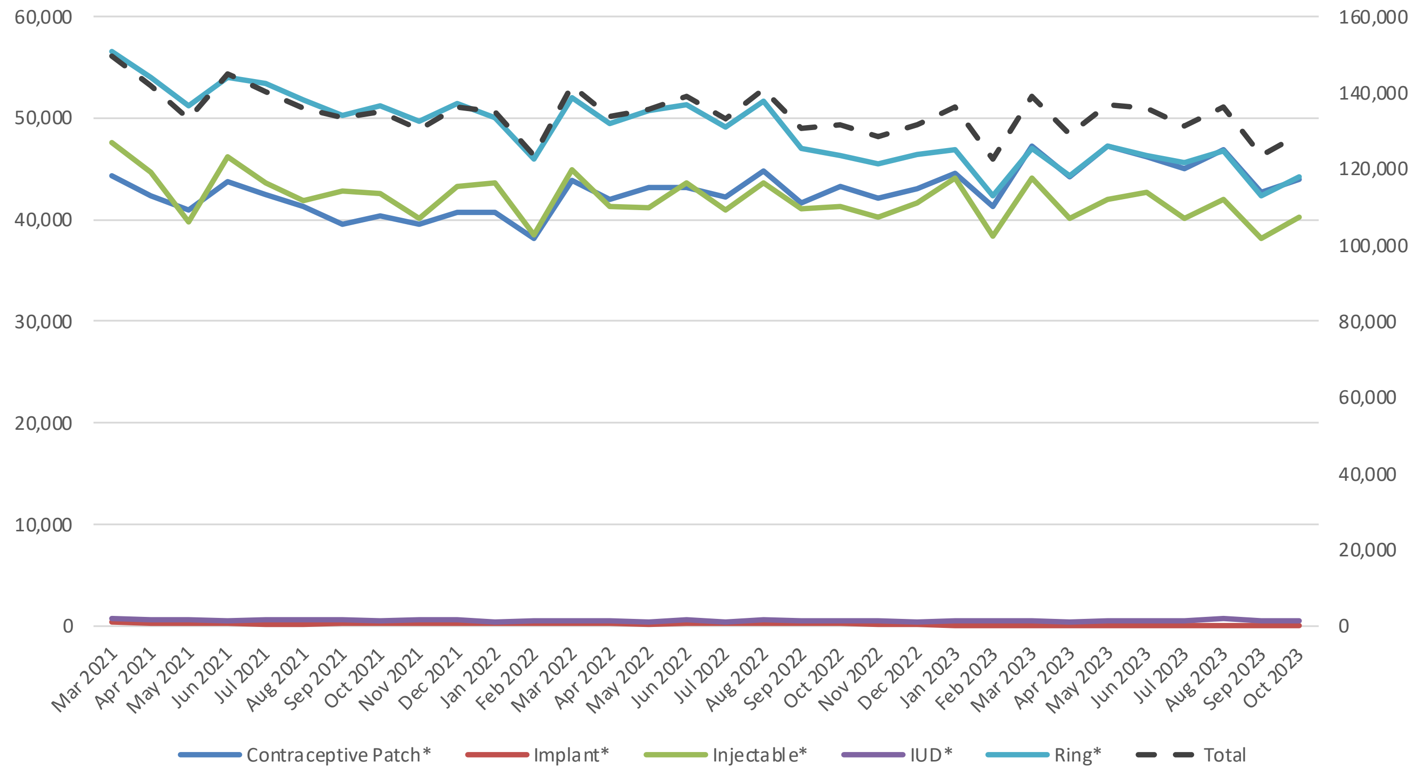

Source: IQVIA National Prescription Audit® PayerTrak™, March 2021-October 2023

© 2024 Qato DM et al. *JAMA Network Open*.

eFigure 5. Trends in Prescriptions Filled in States that Became Most Restrictive vs Comparison States (Excluding Iowa and Wisconsin), March 2021 to October 2023[a,b]

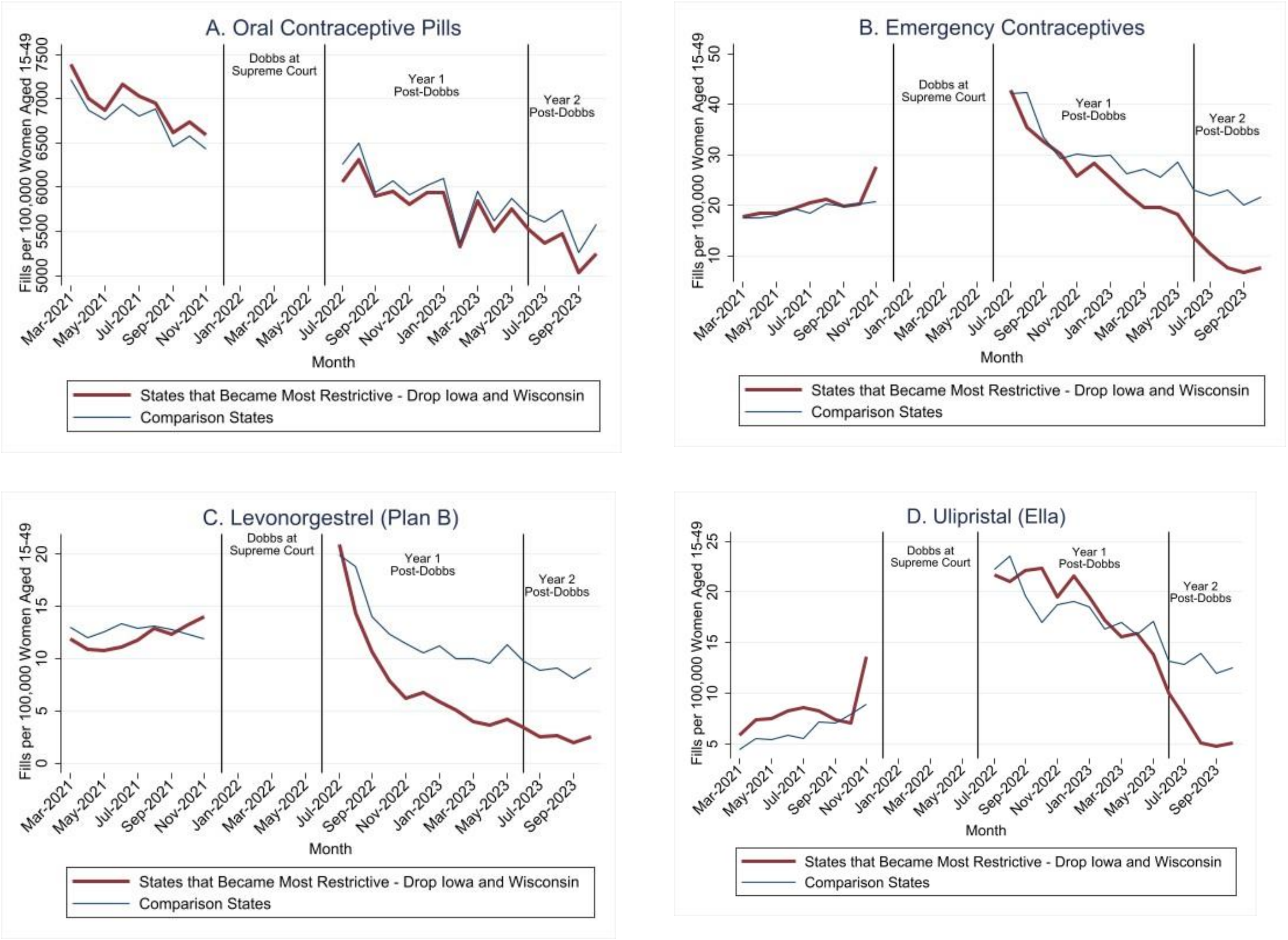

Source: IQVIA National Prescription Audit® (NPA) and IQVIA PayerTrack™

[a] States that Became Most Restrictive: AL, AR, MS, MO, OK, SD, TX, ID, LA, WV, KY, TN; Comparison States (Medium Restrictiveness): FL, KS, PA, UT, DE, MI, MT, NV, NH, RI, VA, WY

[b] Abortion Categories based on authors' analyses of state policy categories provided by the Guttmacher Institute

© 2024 Qato DM et al. *JAMA Network Open*.

eTable 4. Changes in Monthly Fills for Oral Contraceptives Per 100 000 Women of Reproductive Age, 15 to 49 Years, in States That Became Most Restrictive vs Comparison States (excluding Iowa and Wisconsin), March 2021 to October 2023

	Monthly Fills per 100,000 Women of Reproductive Age, mean		Regression Analysis		
**Oral Contraceptive Pills**	**Most Restrictive States[a,b]**	**Comparison States[a,b]**	**Difference-in-differences estimate for monthly fills per capita (95% CI)**	**% Change[c]**	***P*-value**
Pre-*Dobbs* (March 2021 - November 2021)	6927.4	6773.4	Reference		
Post-*Dobbs* (July 2022-October 2023)	5687.8	5843.7	-309.9 (-533.3 to -24.8)	-4.47	0.03
July 2022- June 2023	5823.1	5941.5	-272.3 (-488.8 to -4.6)	-3.93	0.05
July 2023-October 2023	5281.8	5550.5	-422.6 (-711.0 to -86.8)	-6.10	0.02
**Emergency Contraceptives**					
Pre-*Dobbs* (March 2021 - November 2021)	20.3	19.1	Reference		
Post-*Dobbs* (July 2022-October 2023)	21.6	28.4	-8.0 (-18.7 to 1.5)	-39.3	0.09
July 2022- June 2023	26.1	30.6	-5.7 (-18.1 to 6.4)	-28.2	0.30
July 2023-October 2023	8.1	21.6	-14.8 (-29.0 to -6.2)	-72.7	0.000
**Levonorgestrel (Plan-B)**					
Pre-*Dobbs* (March 2021 - November 2021)	12.1	12.7	Reference		
Post-*Dobbs* (July 2022-October 2023)	6.4	11.5	-4.5 (-9.4 to -0.6)	-37.5	0.03
July 2022- June 2023	7.8	12.4	-4.1 (-9.0 to -0.3)	-34.0	0.03
July 2023-October 2023	2.4	8.8	-5.8 (-10.9 to -1.9)	-48.1	0.01
**Ulipristal (Ella)**					
Pre-*Dobbs* (March 2021 - November 2021)	8.2	6.4	Reference		
Post-*Dobbs* (July 2022-October 2023)	15.2	16.9	-3.5 (-12.5 to 5.1)	-42.0	0.47
July 2022- June 2023	18.4	18.2	-1.6 (-10.9 to 9.4)	-19.8	0.75
July 2023-October 2023	5.7	12.8	-9.0 (-20.7 to -1.7)	-108.8	0.003

Source: IQVIA National Prescription Audit® (NPA) and IQVIA PayerTrack™, March 2021-October 2023
a States that Became Most Restrictive: AL, AR, MS, MO, OK, SD, TX, ID, LA, WV, KY, TN; Comparison States (Medium Restrictiveness): FL, KS, PA, UT, DE, MI, MT, NV, NH, RI, VA, WY.
b Abortion Categories based on authors' analyses of state policy categories provided by the Guttmacher Institute
c Percent change is calculated as (DiD estimate/pre-Dobbs mean)*100

© 2024 Qato DM et al. *JAMA Network Open*.
