# Supplementary material for: Use of Oral and Emergency Contraceptives After the US Supreme Court’s Dobbs Decision
Source: JAMA Netw Open. 2024 Jun 26;7(6):e2418620. doi: 10.1001/jamanetworkopen.2024.18620 (PMC11208973; doi:10.1001/jamanetworkopen.2024.18620)
Supplement: Supplement 2. — Data Sharing Statement [file jamanetwopen-e2418620-s002.pdf]

Data Sharing Statement

Qato. Changes in Use of Oral and Emergency Contraceptives After the US Supreme Court's Dobbs Decision. *JAMA Netw Open*. Published June 26, 2024.
doi:10.1001/jamanetworkopen.2024.18620

Data
Data available: No

Additional Information
Explanation for why data not available: These data are proprietary and can be licensed directly from IQVIA
